# Supplementary material for: Inverse design of chiral functional films by a robotic AI-guided system
Source: Nat Commun. 2023 Oct 4;14:6177. doi: 10.1038/s41467-023-41951-x (PMC10551020; doi:10.1038/s41467-023-41951-x)
Supplement: Supplementary file 1 — Supplementary Information [file 41467_2023_41951_MOESM1_ESM.pdf]

Supplementary Information

Inverse design of chiral functional films by a robotic AI-guided system

Yifan Xie[1†], Shuo Feng[1†], Linxiao Deng[2], Aoran Cai[1], Liyu Gan[1], Zifan Jiang[1], Peng Yang[1], Guilin Ye[3], Zaiqing Liu[1], Li Wen[1], QingZhu[1], Wanjun Zhang[3], Zhanpeng Zhang[1], Jiahe Li[1], Zeyu Feng[1], Chutian Zhang[1], Wenjie Du[1], Lixin Xu[2], Jun Jiang[1*], Xin Chen[4*] and Gang Zou[1*]

[1]Key Laboratory of Precision and Intelligent Chemistry, School of Chemistry and Materials Science, University of Science and Technology of China, Hefei, Anhui 230026, China
[2]State Key Laboratory of Particle Detection and Electronics, Department of Optics and Optical Engineering, University of Science and Technology of China, Hefei, Anhui 230026, China
[3]Hefei JiShu Quantum Technology Co. Ltd., Hefei 230026, China
[4]Suzhou Laboratory, No. 388, Ruoshui Street, SIP, Jiangsu 215123, China

[†]These authors contributed equally.
[*]Email: jiangj1@ustc.edu.cn; mail.xinchen@gmail.com; gangzou@ustc.edu.cn;

<u>Contents</u>

Supplementary Tables 1-7

Supplementary Figures 1-36

Supplementary References

Supplementary Figures

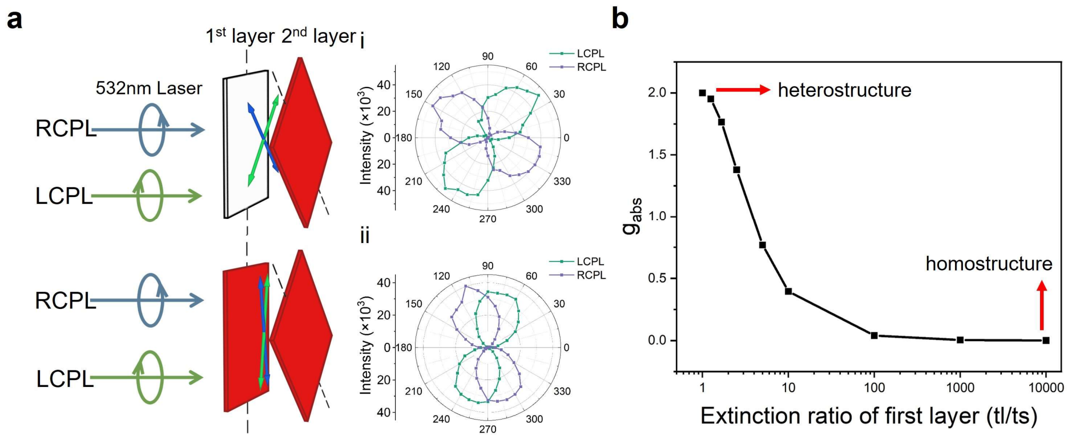

Supplementary Fig. 1 | Schematic illustration of highly chiroptical active hetero-structured bilayer construction. (**a**) Illustration of the polarization state of left-handed/ right-handed circularly polarized light passing through the first layer of the hetero-structured bilayer (i: transparent film, ii: dyed films with polarization). (**b**) The Jones matrix of the hetero-structured bilayer and the variation of dissymmetry factor g_{abs} with the extinction ratio of the second layer.

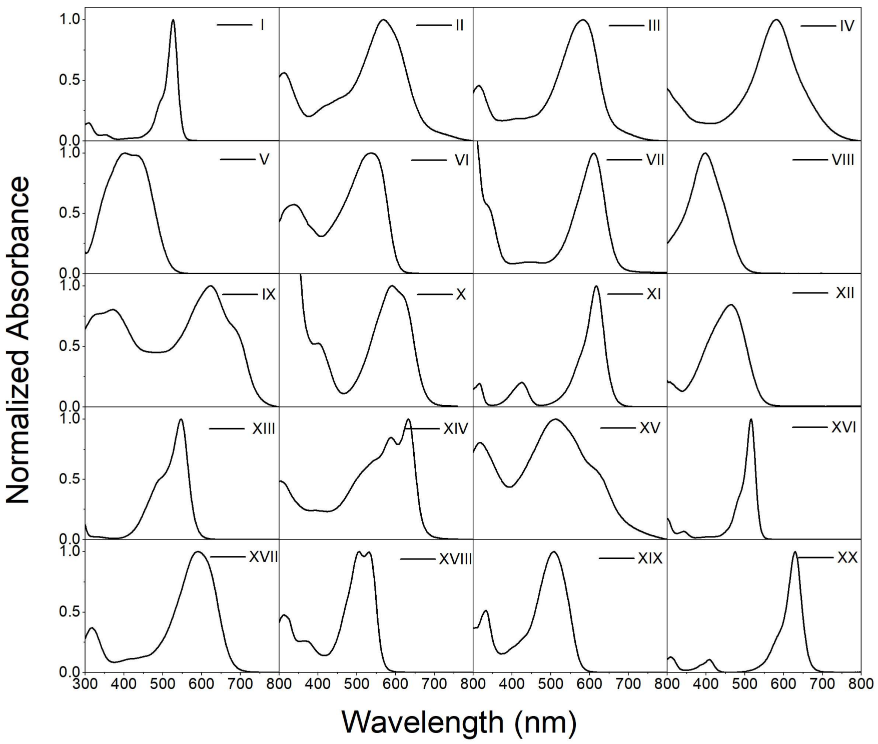

Supplementary Fig. 2 | The absorption spectra of 20 dyes. (I: erythrosine, II: direct blue 2, III: direct blue 6, IV: direct blue 71, V: basic orange 2, VI: congo red, VII: indigo carmine, VIII: Sirius yellow, IX: aizendirectgreenbh, X: reactive blue 4, XI: malachitegreen, XII: methyl orange, XIII: basicfuchisin, XIV:direct purple 1, XV: direct red 13, XVI: eosin y, XVII: amaniskybluer, XVIII: azophloxine, XIX: new coccine, XX: erioglaucinediammoniumsalt).

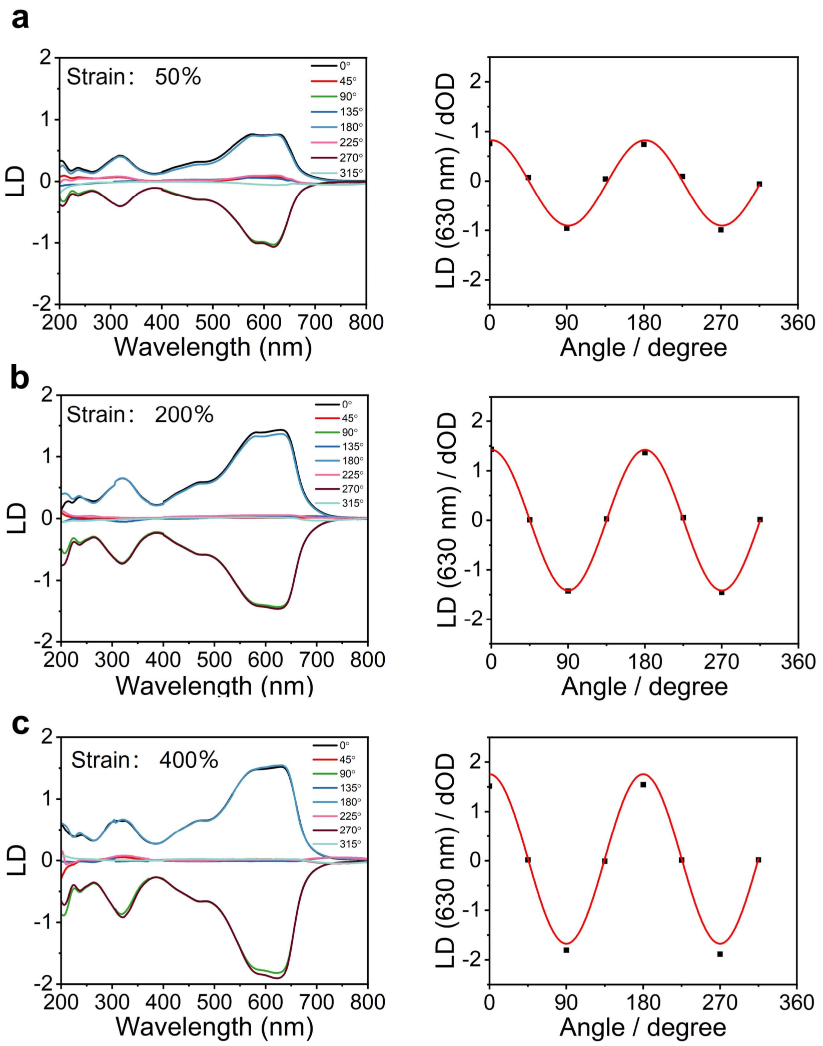

Supplementary Fig. 3 | The linear dichroism (LD) spectra of the oriented film. The LD spectra of the dyed film (80 μm thickness) with dye 7 were measured under different strains (50%(a), 200%(b), 400%(c)) while rotating around the optical path at different angles.

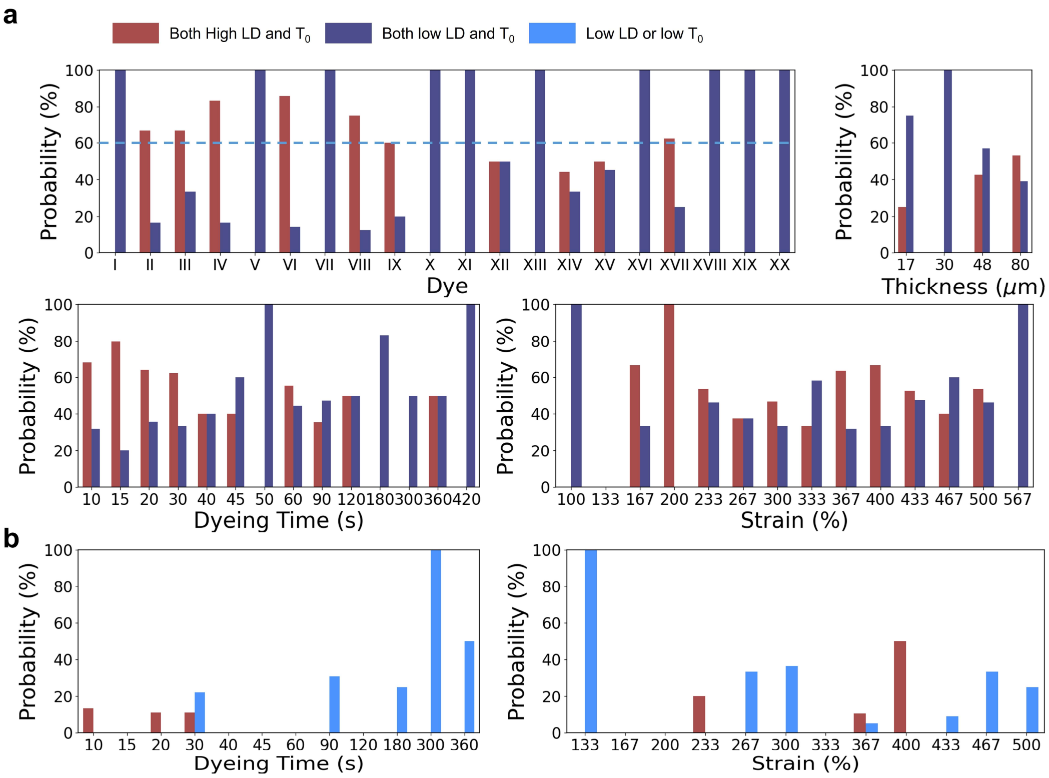

Supplementary Fig. 4 | Results of the clustering-screening for dyed films. (**a**) P_{high} and P_{low} of parameter values for dyed film in the first round screening (n =118). A value is removed when its P_{low} is larger than 60% for dye molecule and thickness. For strain and dyeing time, the range of parameter is narrowed when the boundary values have large P_{low}. This step screens 10 dye molecules and 3 thickness values, and narrows the ranges of both strain and dyeing time. (**b**) P_{high} and P_{low} of parameter values for dyed film in the second round screening (n = 80), which only performs on the strain and dyeing time.

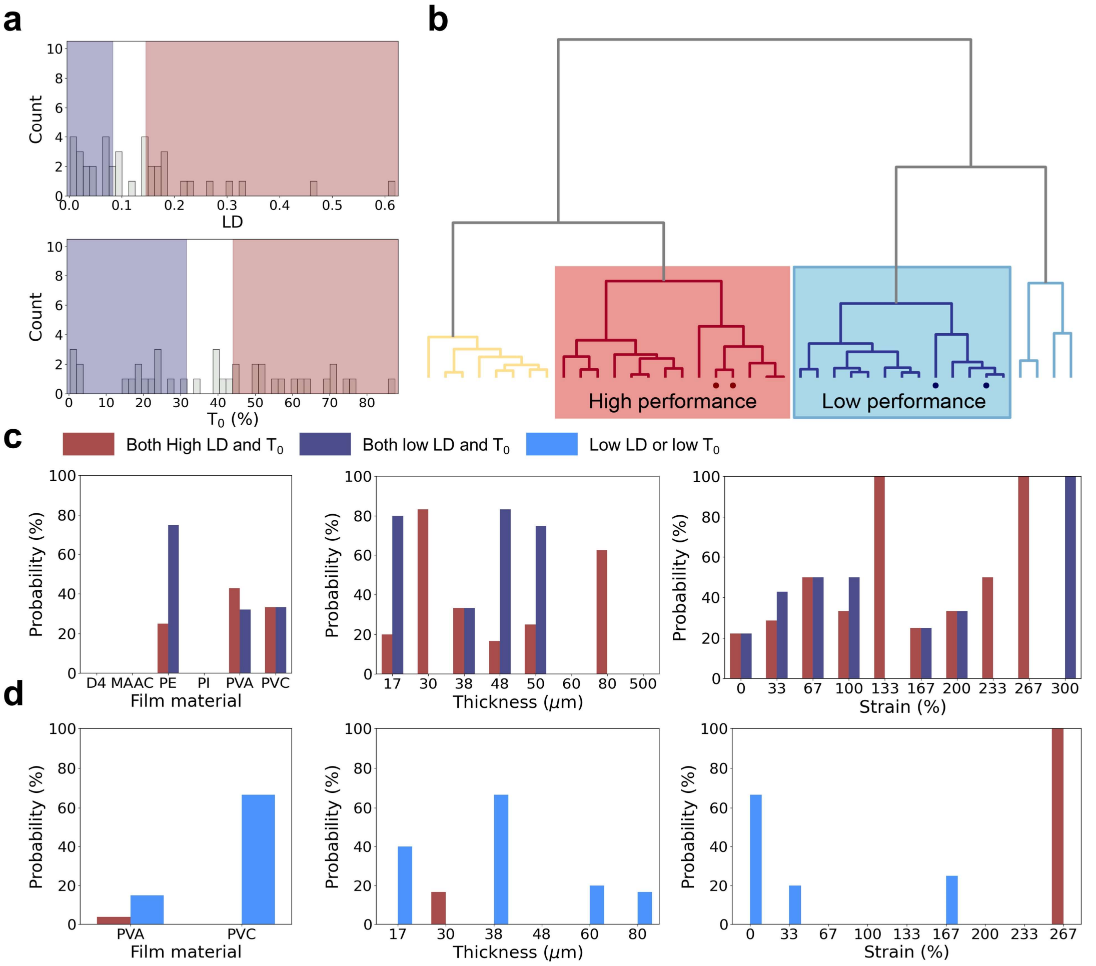

Supplementary Fig. 5 | Results of the clustering-screening for transparent films. (**a**) Histogram of LD and T_0 for transparent films. (**b**) after hierarchical clustering, transparent films form four clusters with one high performance (highlighted in red shading) and one low performance (highlighted in blue shading). (**c**) P_{high} and P_{low} of parameter values for transparent film in the first round screening (n=39). (**d**) P_{high} and P_{low} of parameter values for transparent film in the second round screening (n=30).

Supplementary Table 1. Estimated numbers of parameter combinations before and after the clustering-screening.

**Structure/Process parameters**		**Number (and range) of values**		
		**Before screening**	**After 1st round**	**After 2nd round**
**Dyed film**	**Dye (Absorption)**	20	10	10
	**Dyeing time (Greyscale)**	84 (0 s - 420 s)	78 (0 s - 390 s)	40 (0 s - 200 s)
	**Thickness**	4	1	1 (80 μm)
	**Strain**	18 (0% - 600%)	12 (133% - 533%)	1 (400%)
**Transparent film**	**Materials Selection**	6	2	1 (PVA)
	**Thickness**	6	6	5
	**Strain**	60 (0% - 300%)	55 (0% - 275%)	50 (20% - 270%)
**Twist angle**		2	2	2
**Total combinations**		5.23×10^{8}	1.24×10^{7}	2.00×10^{5}

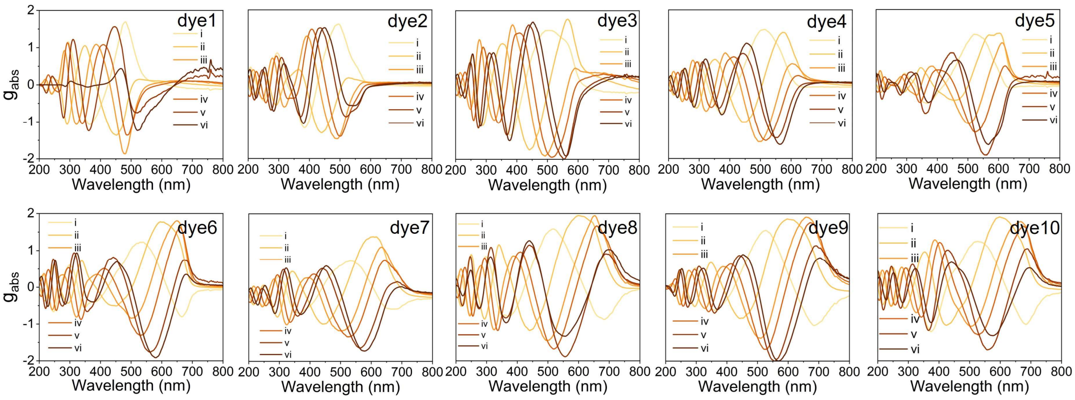

Supplementary Fig. 6 | Strain dependence of chiroptical activity of the composite films. The dissymmetry factor g_{abs} spectra of 10 dyes at various strain (i:67%, ii:80%, iii:100%, iv:133%, v:167%, vi:200%) under the condition that the thickness of retardation film (PVA) is 80 μm and the greyscale is 5.

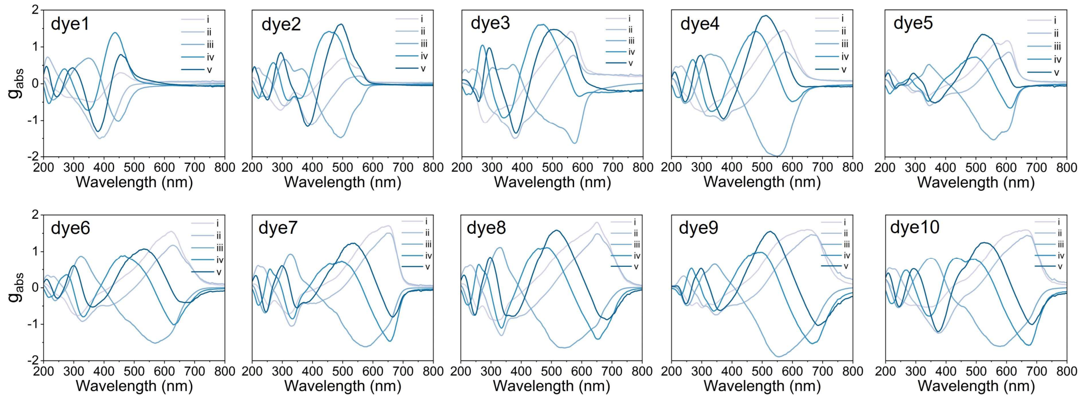

Supplementary Fig. 7 | Thickness dependence of chiroptical activity of the composite films. The dissymmetry factor g_{abs} spectra of 10 dye molecules at different thickness (i:17 μm, ii:30 μm, iii:48 μm, iv:60 μm, v:80 μm) under the condition that the greyscale is 5 and the strain is 100%.

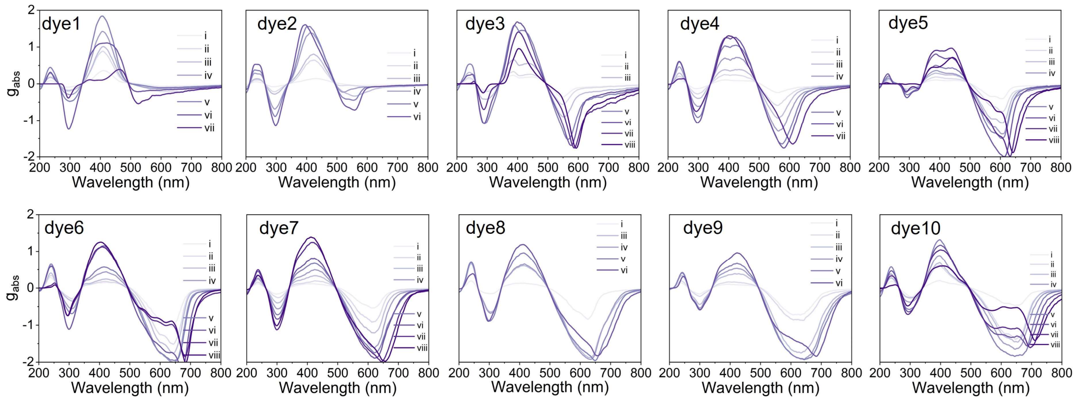

Supplementary Fig. 8 | Dye molecule concentration dependence of chiroptical activity of the composite films. The dissymmetry factor g_{abs} spectra of 10 dye molecules at different greyscales (i:greyscale1, i:greyscale2, iii:greyscale3, iv:greyscale4, v:greyscale5, vi:greyscale6, vii:greyscale7, viii:greyscale8) under the condition that the thickness of retardation film (PVA) is 48 μm and the strain is 100%.

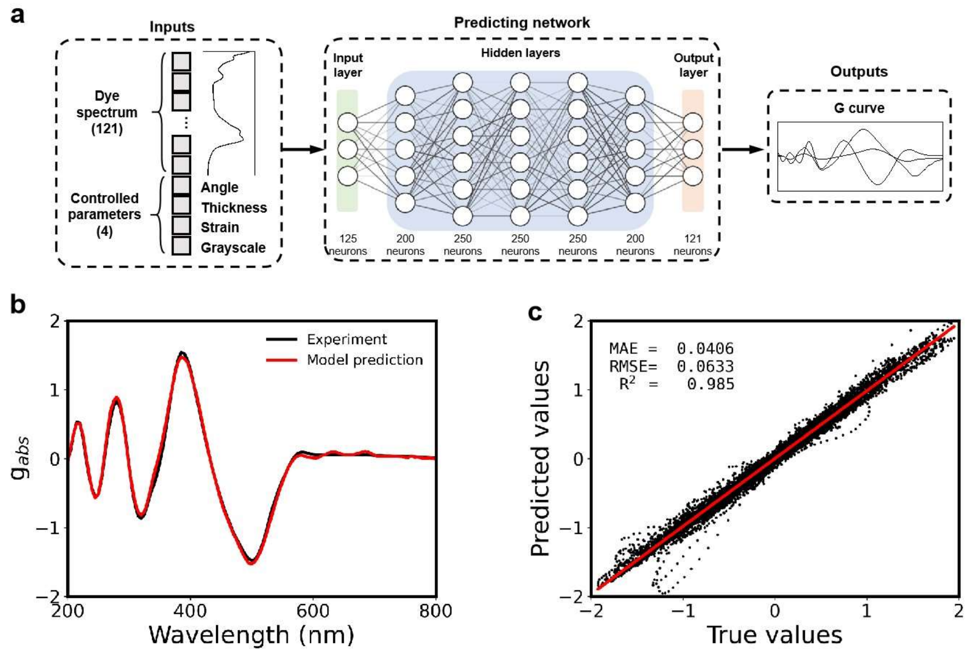

Supplementary Fig. 9 | Schematic illustration of the forward prediction model and its performance. (**a**) Structure of the neural network model used for forward prediction. (**b**) Comparison of model prediction and experiment for a typical CD spectrum in the testing set. (**c**) Comparison of all spectra in the testing set.

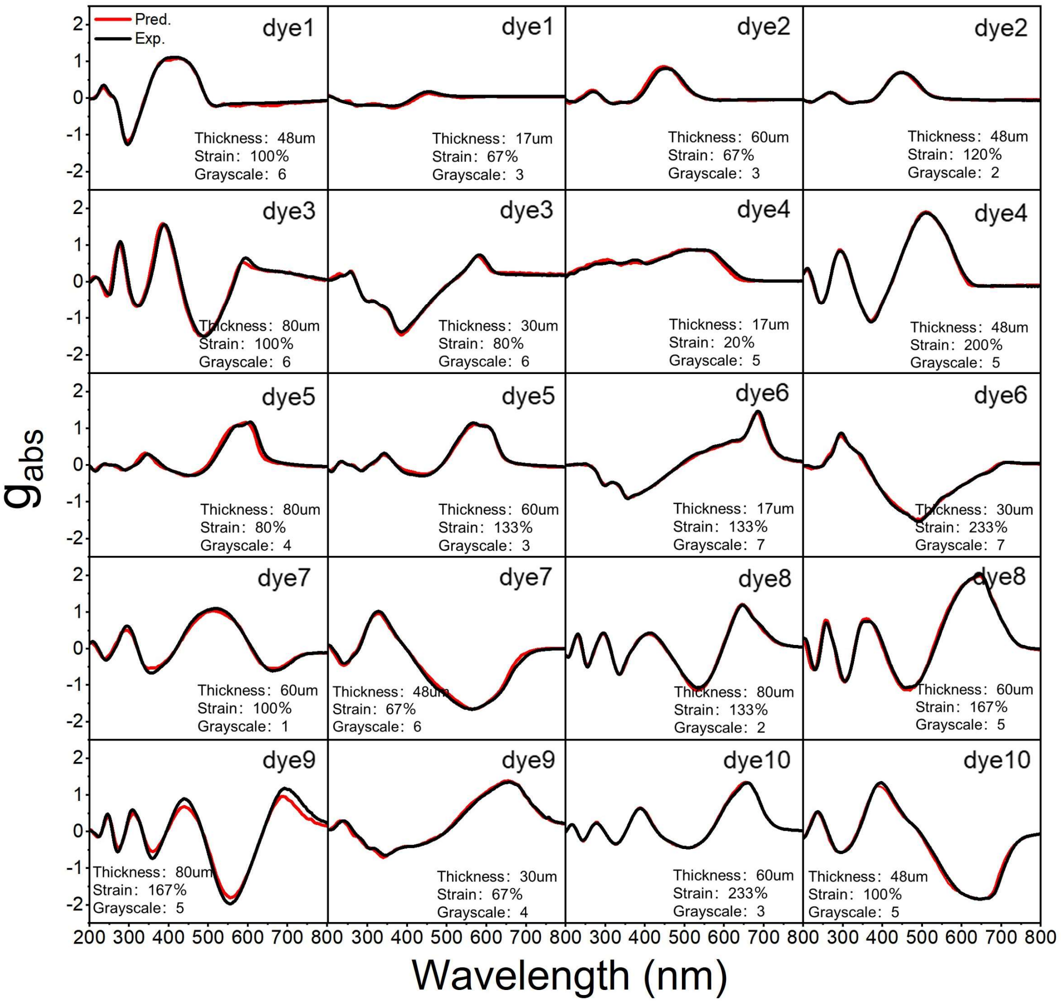

Supplementary Fig. 10 | Comparison between predicted and experimental g_{abs} spectra. Predicted vs. experimentally measured dissymmetry factor g_{abs} spectra of 20 thin films with a twist angle of 45° prepared using randomly selected parameters.

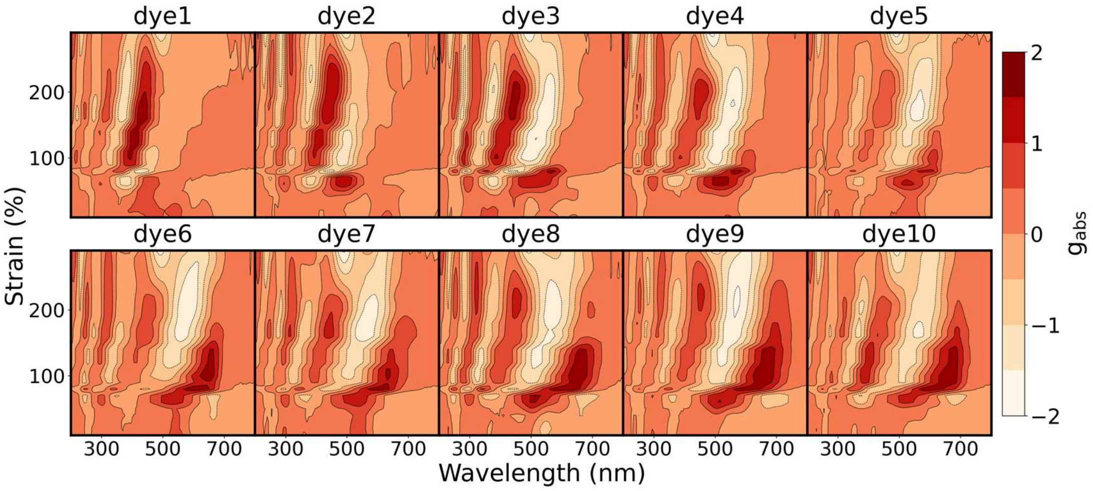

Supplementary Fig. 11 | Strain dependence of model predicted chiroptical activity of the composite films. The model predicted dissymmetry factor g_{abs} values of 10 dyes at different strain under the condition of thickness 80 µm, greyscale 5 and angle 45°. With the increment of strain, the peaks on g_{abs} spectra move to the direction of large wavelength.

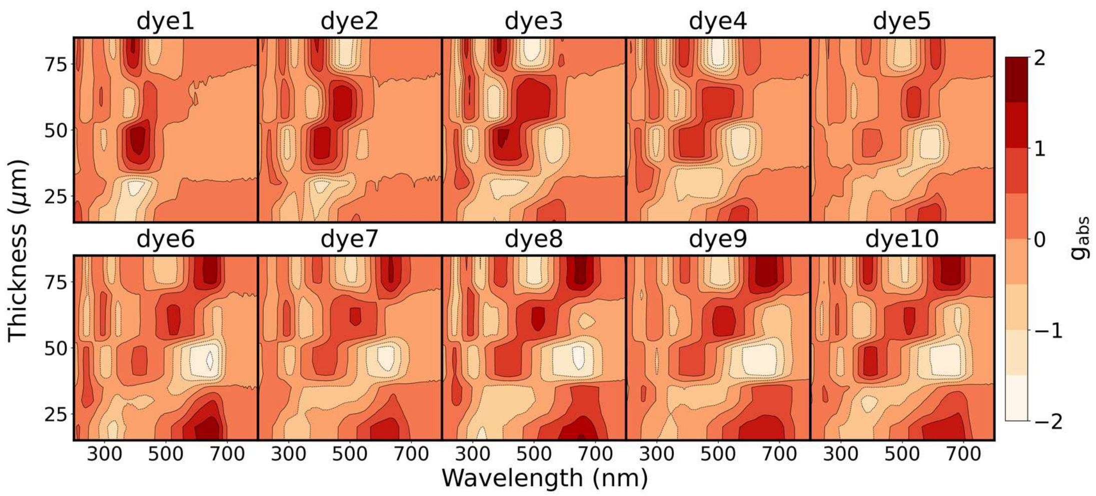

Supplementary Fig. 12 | Thickness dependence of model predicted chiroptical activity of the composite films. The model predicted dissymmetry factor g_{abs} values of 10 dyes at different thickness under the condition of strain 100%, greyscale 5 and angle 45°. The distance between peaks and troughs of g_{abs} spectra decreases with the increment of thickness.

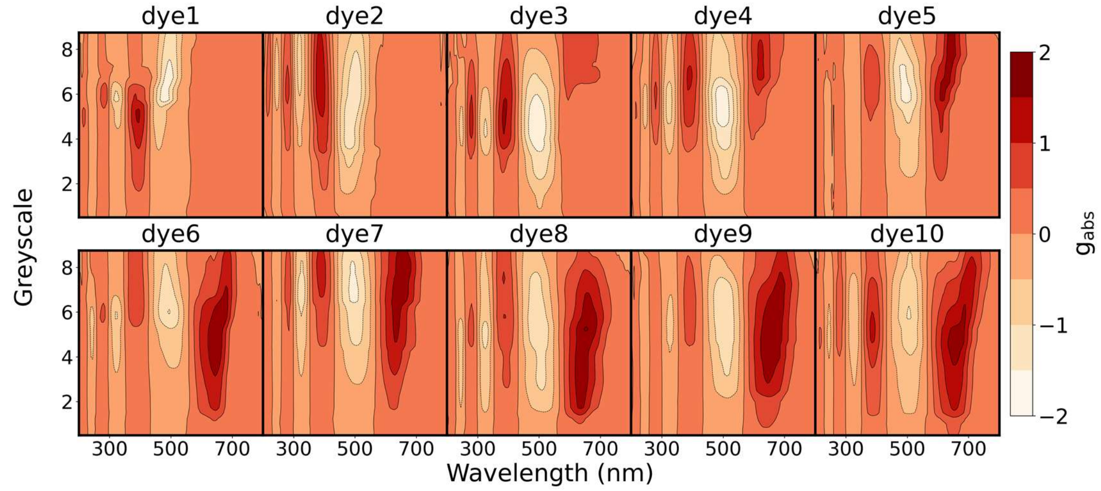

Supplementary Fig. 13 | Greyscale dependence of model predicted chiroptical activity of the composite films. The model predicted dissymmetry factor g_{abs} values of 10 dyes at different greyscale under the condition of thickness 80 μm, strain 100% and angle 45°. With the increment of greyscale, g_{abs} increase first and then decrease, while the waveform of g_{abs} spectra keeps largely unchanged.

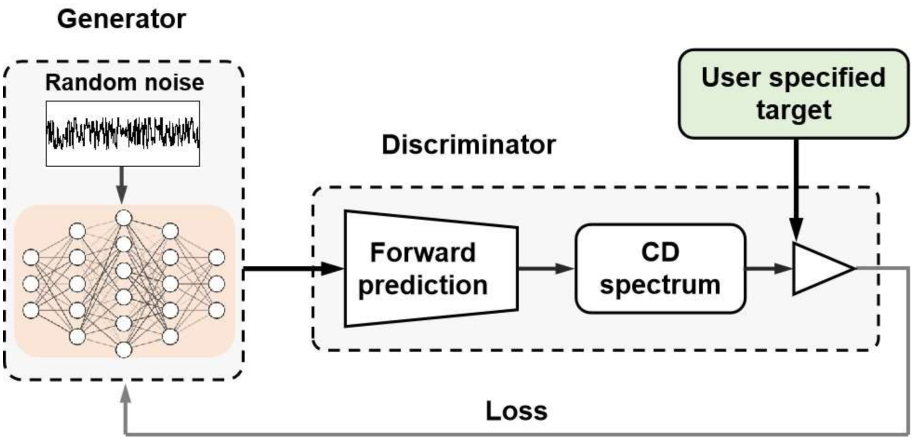

Supplementary Fig. 14 | Overall architecture of the reverse model. A generator produces a random set of parameters. After being restructured into spectrum embedded descriptors, they are used as input parameters for the forward prediction model, which produces a circular dichroism (CD) spectrum based on the machine learned quantitative structure-spectrum-activity relationship (QSSAR). The key spectral features are compared with user-specified target properties. The loss (difference between predicted properties and target ones) is minimized by recursive optimization based on the Adam algorithm.

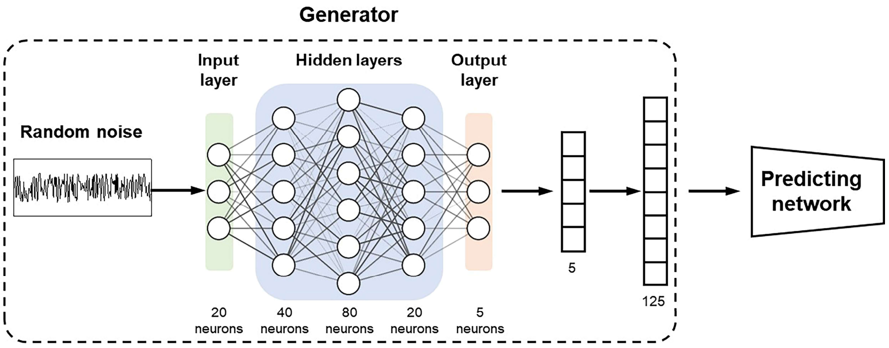

Supplementary Fig. 15 | Schematic illustration of the generator in the reverse model. The generator is a fully connected neural network consisting of a 20-nodes input layer, a 5-nodes output layer, and 3 hidden layers whose numbers of nodes are 40, 80, and 20, respectively. All settings of the generator are the same as the forward network except the batch size (16) and the number of epochs (50). To connect the generator with the forward network, the first number of the generator's output, who represents the species of dyes, is converted into a one-hot vector, and then multiplies with a 10×121 matrix where each row represents the absorption spectrum of one dye. As a result, the species of dyes are transformed into the corresponding absorption.

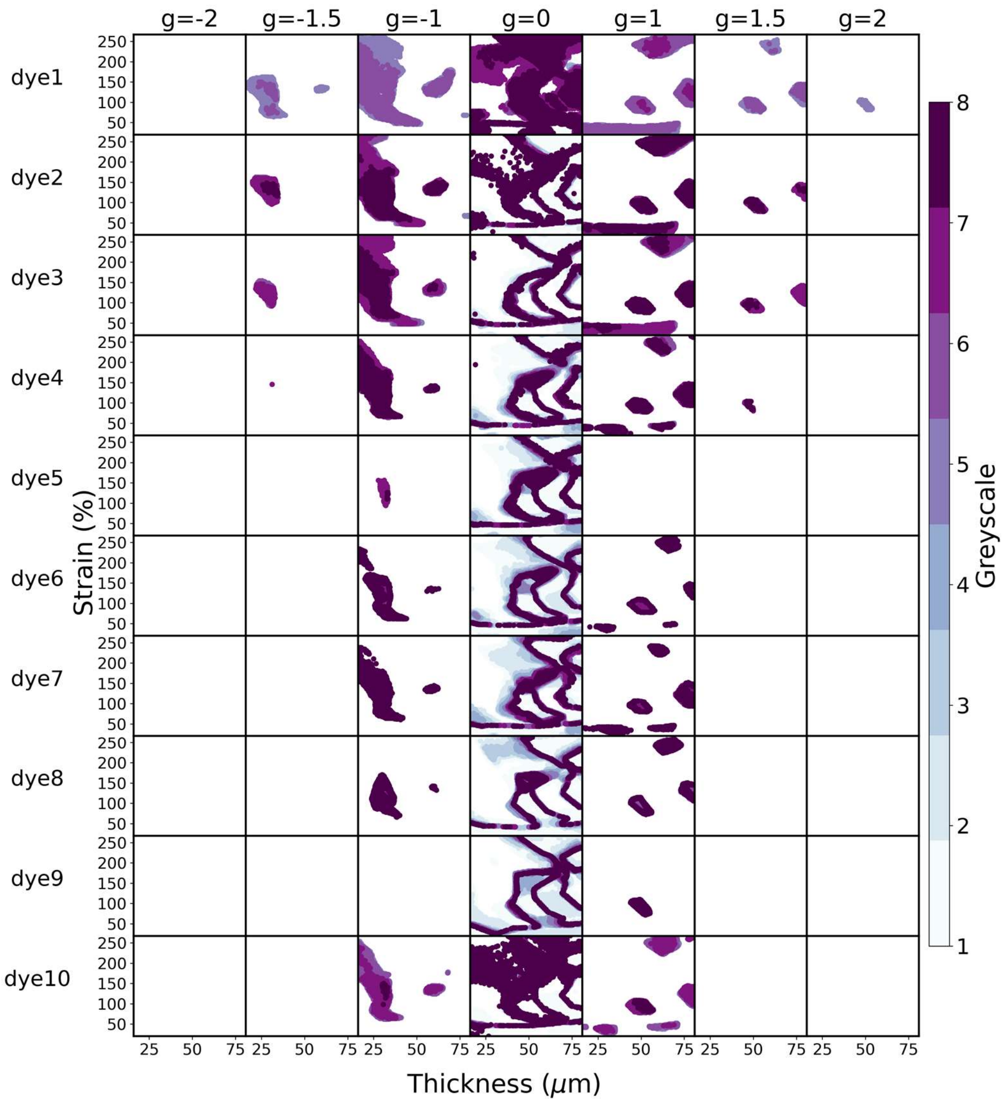

Supplementary Fig. 16 | Distributions of available answers for a target dissymmetry factor g_{abs} value. Results of virtual high-throughput screening of designed parameters for different target g_{abs} at 400 nm. The angle is fixed as 45°. When the absolute value of g_{abs} is near 0, a large amount of parameter combinations can satisfy the target. With the increment of g_{abs}'s absolute value, the range of parameters keeps shrinking. For some dyes, it becomes impossible to find a set of controlled parameters at high g_{abs}. As a result, the highest g_{abs} value limits the dye species and control parameters in one or several small ranges, in which we can choose suitable parameters for experiments.

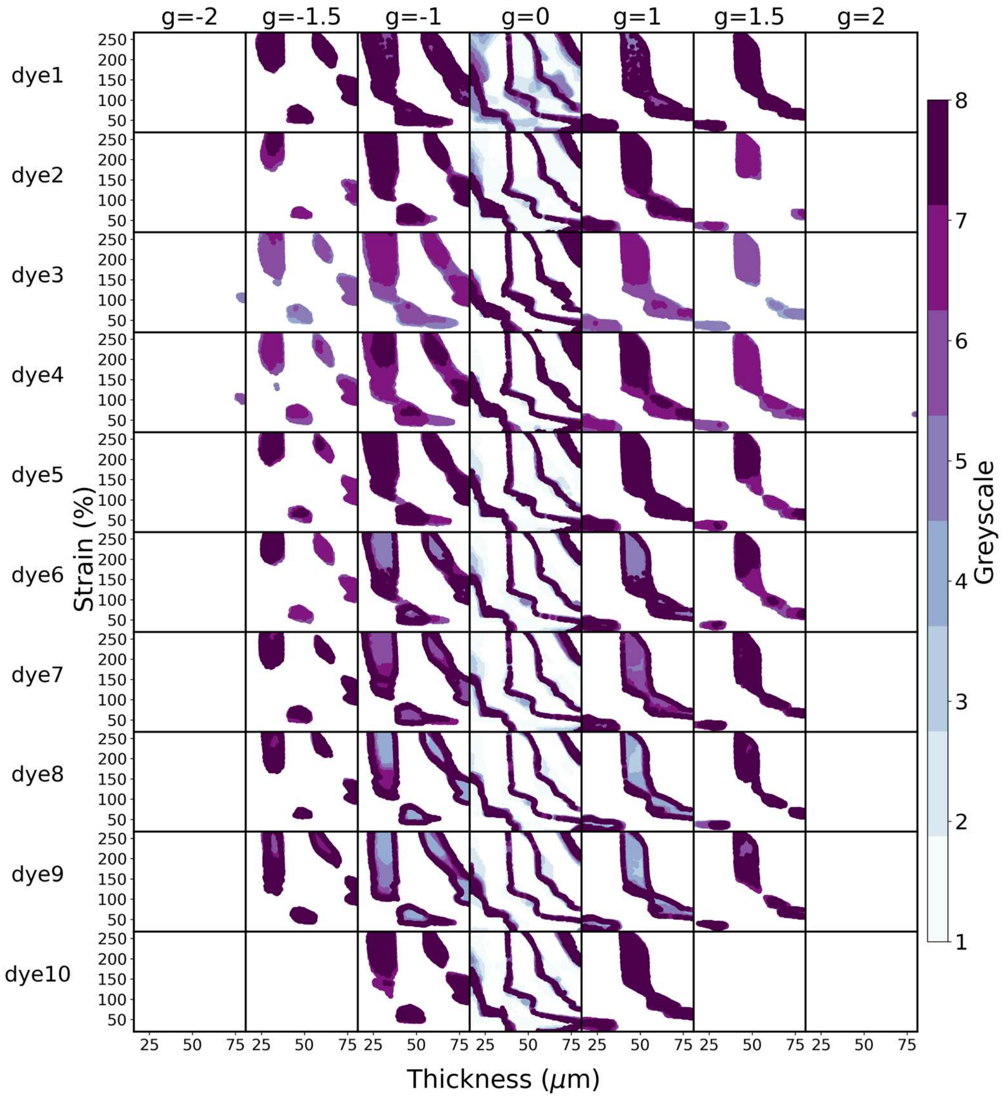

Supplementary Fig. 17 | Distributions of available answers for a target dissymmetry factor g_{abs} value. Results of virtual high-throughput screening of designed parameters for different target g_{abs} at 500 nm.

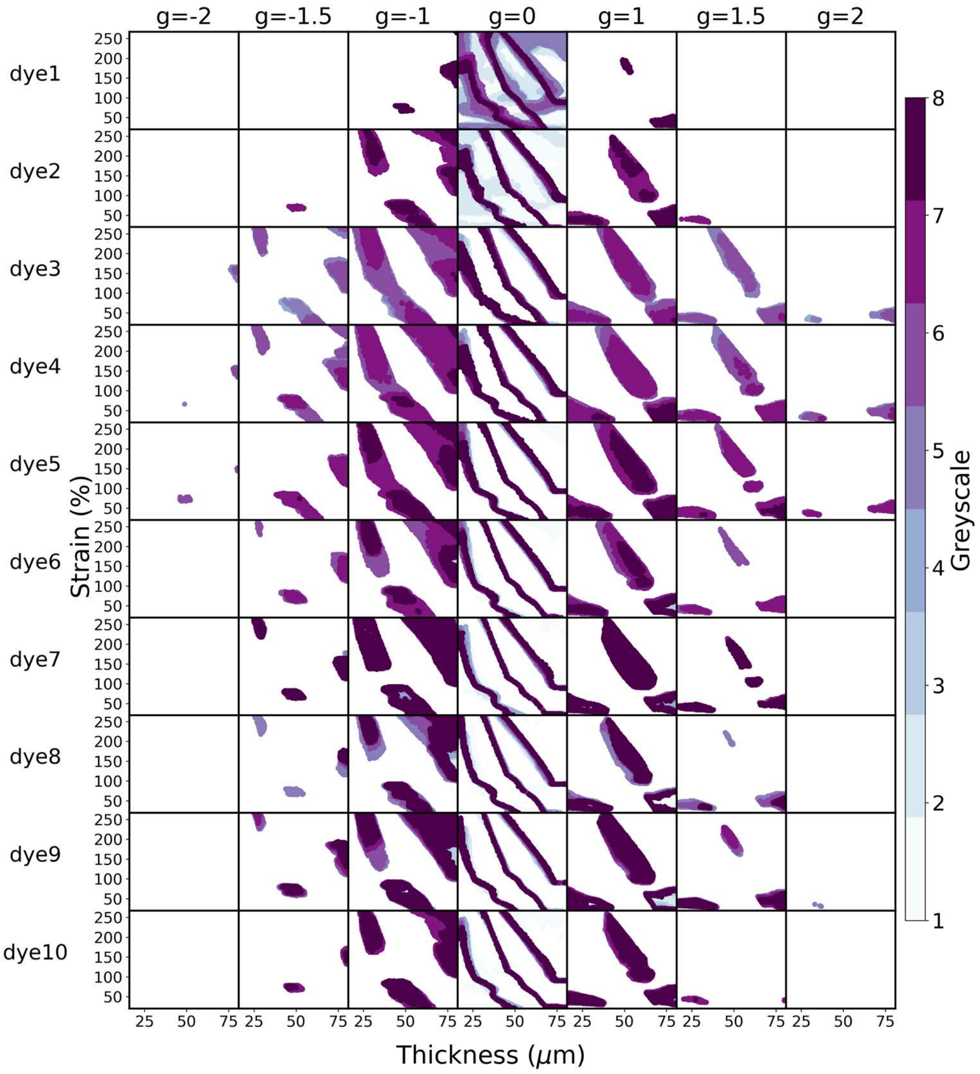

Supplementary Fig. 18 | Distributions of available answers for a target dissymmetry factor g_{abs} value. Results of virtual high-throughput screening of designed parameters for different target g_{abs} at 531 nm.

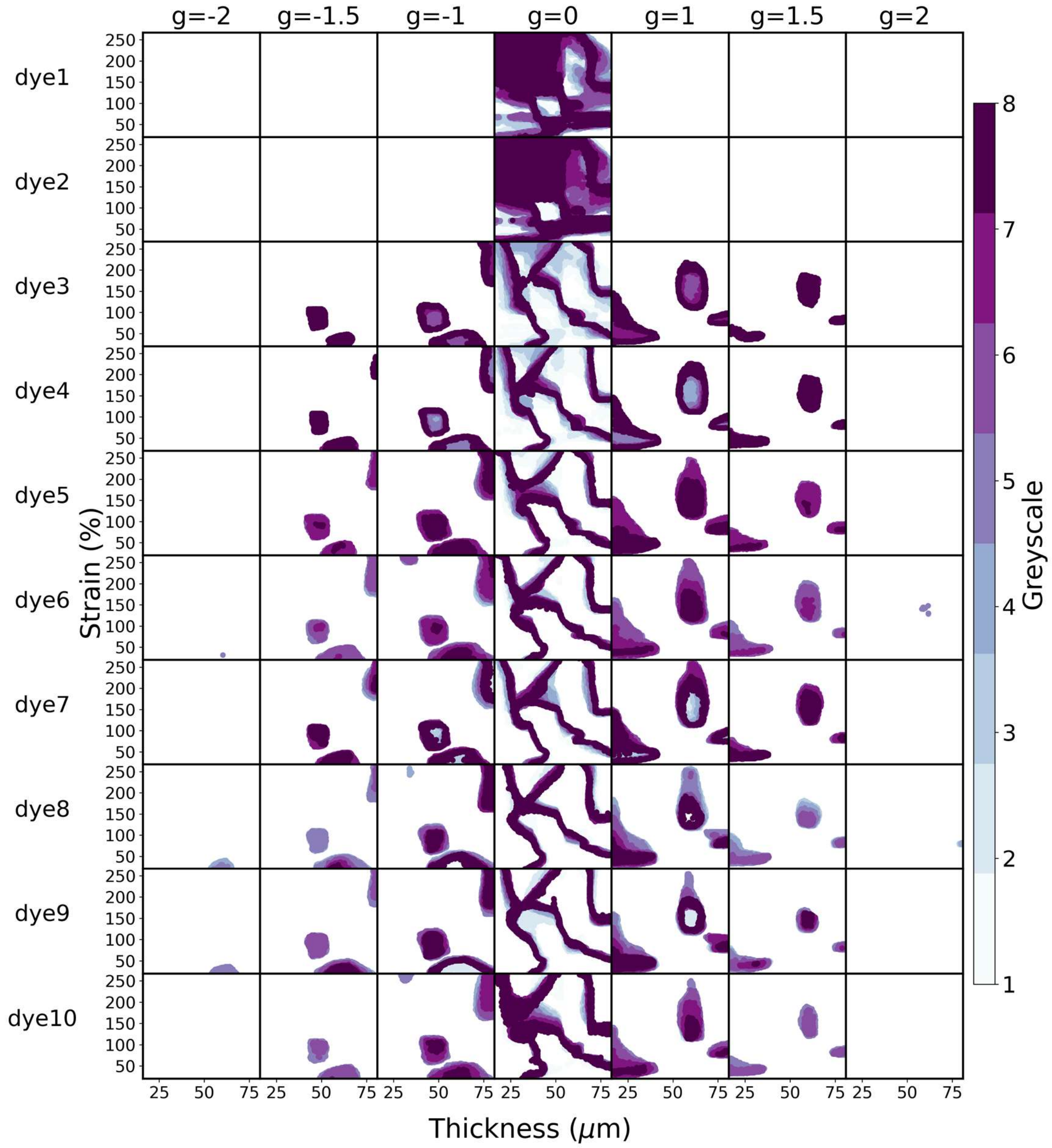

Supplementary Fig. 19 | Distributions of available answers for a target dissymmetry factor g_{abs} value. Results of virtual high-throughput screening of designed parameters for different target g_{abs} at 600 nm.

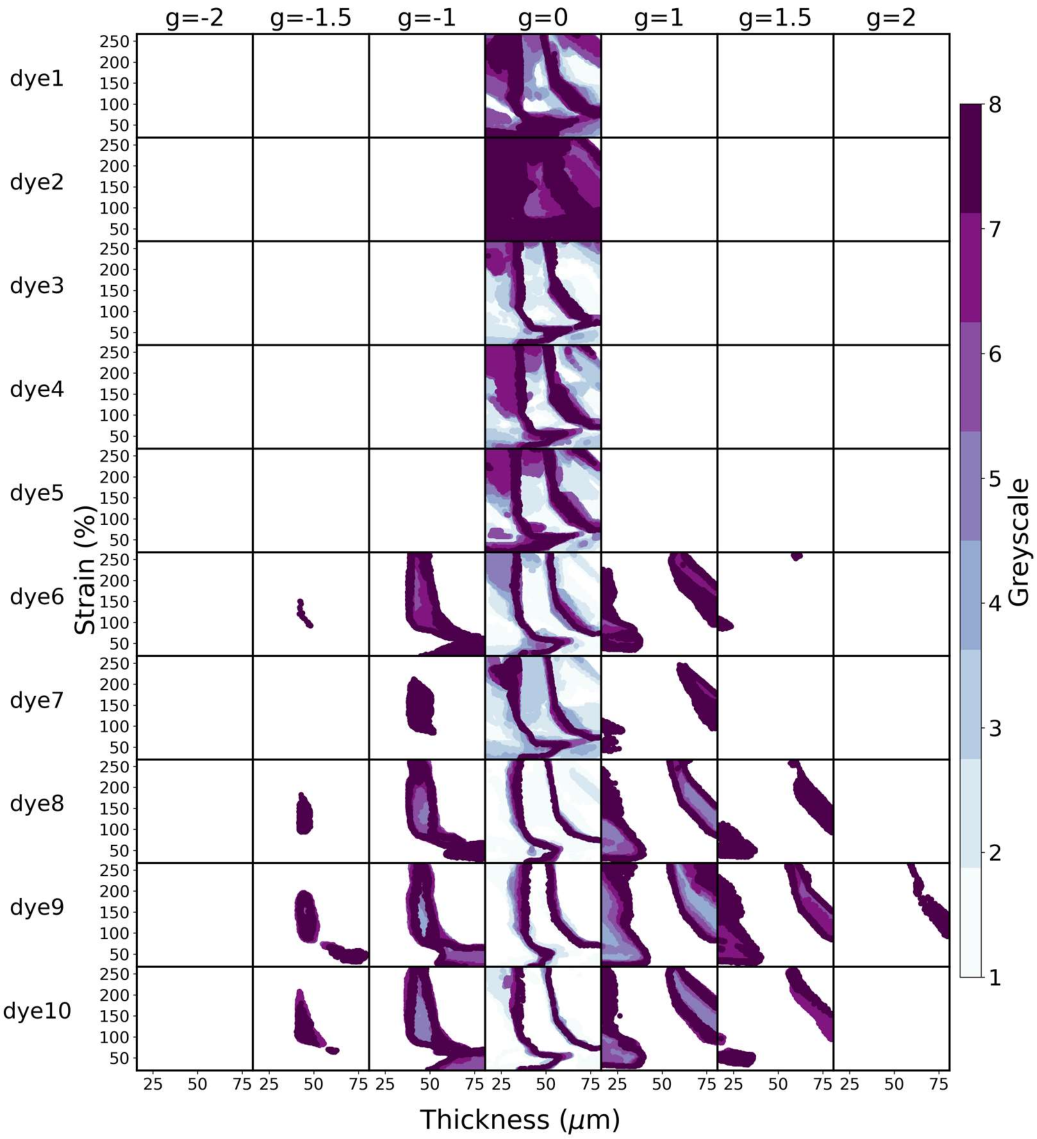

Supplementary Fig. 20 | Distributions of available answers for a target dissymmetry factor g_{abs} value. Results of virtual high-throughput screening of designed parameters for different target g_{abs} at 700 nm.

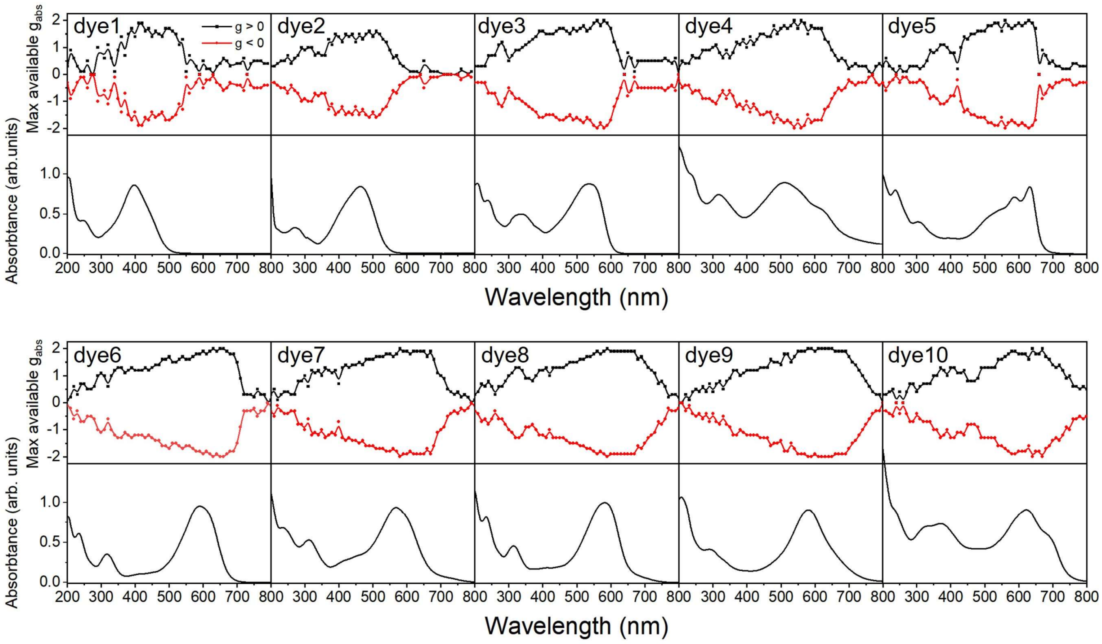

Supplementary Fig. 21 | Wavelength dependence of maximum dissymmetry factor |g_{abs}| values possible for each dye molecule. The wavelength dependence of maximum (black) and minimum (red) accessible g_{abs} values for each dye molecule.

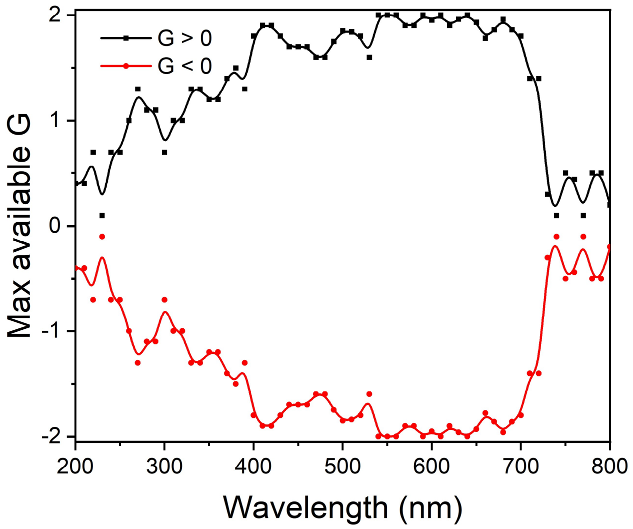

Supplementary Fig. 22 | Wavelength dependence of maximum dissymmetry factor $|g_{abs}|$ values possible for ten dyes. The maximum g_{abs} accessible at different wavelengths using one of the ten dyes.

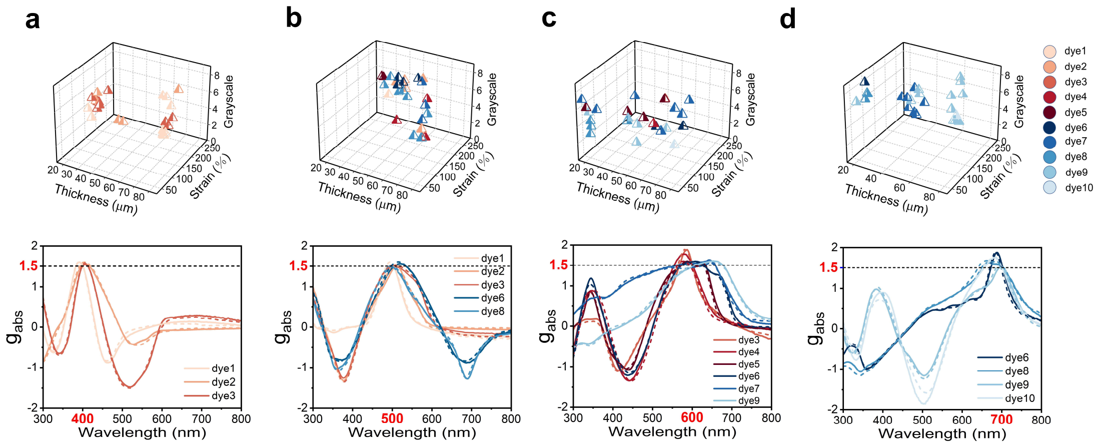

Supplementary Fig. 23 | Examples of experimental realization of inverse design for a target g_{abs}=1.5 at 400 nm (a), 500 nm (b), 600 nm (c),700 nm (d). Fig. 5c in the main text plots the same data in (**b**). The dashed lines are the predicted CD spectra, and the corresponding solid lines are the experimental ones.

Supplementary Table 2. The recipes of the Fig. 2c about experimental realization of inverse design for a target dissymmetry factor g_{abs}=1.5 at 500 nm

Dyes	thickness	strain	greyscale
1	48	132	7
2	45	186	7.2
2	46	220	6.8
2	49	206	6.2
2	80	66	6
2	56	242	2
3	80	68	5
4	59	96	5.3
4	66	192	4
4	77	106	4
5	25	248	5
5	26	250	5
6	46	190	7.7
6	50	160	7.6
6	48	200	7
6	53	238	6
7	80	68	7
7	61	200	7
7	73	132	7
8	75	66	5
8	42	180	7.8
8	51	200	5.4
8	42	218	5.2
8	80	66	5
8	48	200	6
8	28	240	8
8	75	128	8

Supplementary Table 3. The comparison of the dissymmetric factor g_{abs}. (The theoretical limit of dissymmetric factor is ±2.)

structure	$\|g_{abs}\|$	method	year, ref.
Chiral Molecules	**1.44×10^{-3}**	intrinsic	2022, 1
Supramolecular assembly	**4.2×10^{-2}**	intrinsic	2017, 2
Π-Conjugated Systems	**0.87**	intrinsic	2019, 3
Photonic Crystal	**1.6**	extrinsic	2019, 4
Hetero-bilayer film	**1.9**	extrinsic	**This work**

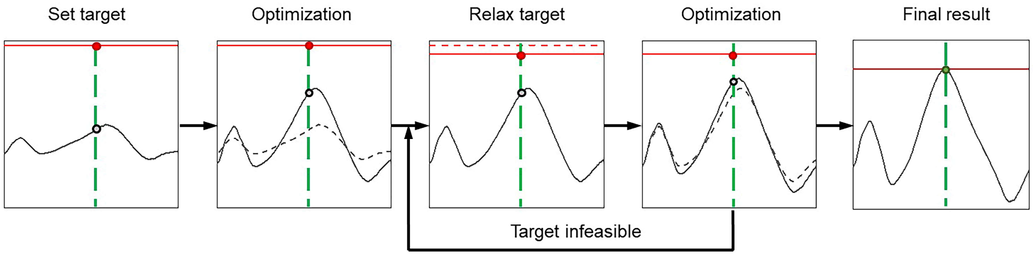

Supplementary Fig. 24 | Schematic of the optimization procedure of design parameters with target relaxation. First, the inverse design network is trained by loss defined with the initial target. If the best designed parameters fail to satisfy the target, a new relaxed target will be set. Then, new parameters are designed, and the performance usually gets better. By repeating the relaxation and inverse design processes, the designed parameters finally reach the best available target.

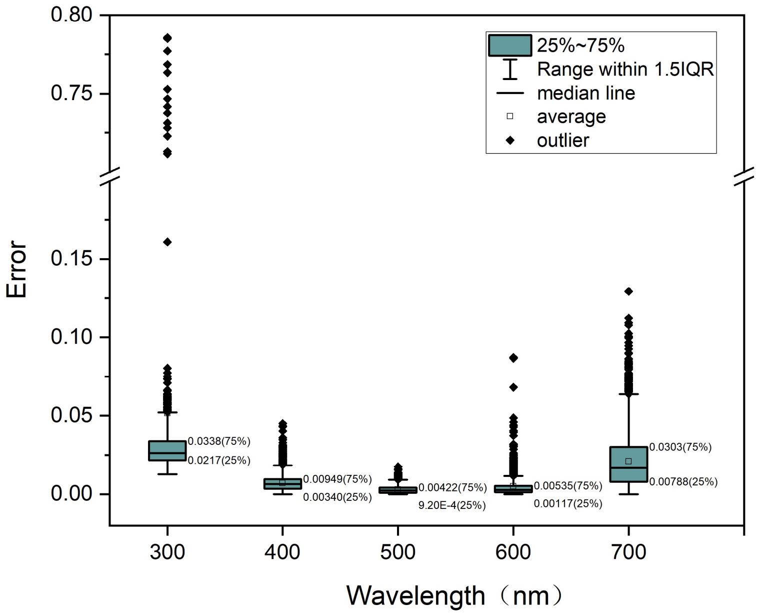

Supplementary Fig. 25 | Comparison of performance of reverse model at different wavelength. The errors represent the differences between optimized dissymmetry factor g values and the target g values. Statistics are performed on 1725, 2501, 5729, 1622 and 2941 points for 300 nm, 400 nm, 500 nm, 600 nm and 700 nm, respectively. As the wavelength increases, the average error shows a trend of first decreasing and then increasing (mean and SEM for 300 nm: 0.05165±0.00287; 400 nm: 0.00702±0.00010; 500 nm: 0.00287±0.00003; 600nm: 0.00489±0.00002; 700 nm: 0.02101±0.00032). In boxplots the lower hinge represents 25% quantile, upper hinge 75% quantile, and center line the median. Whiskers extend 1.5 times interquartile range (IQR).

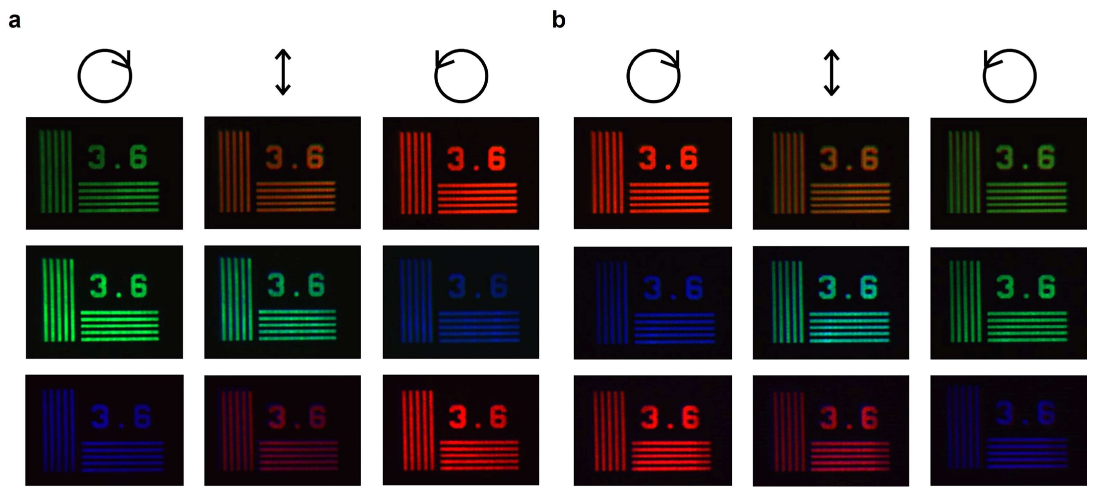

Supplementary Fig. 26 | Color switching ((a) G-R switch, G-B switch, B-R switch and (b) R-G switch, B-G switch, R-B switch) versus incident polarization states for chiroptical activity films (different with the Fig. 7, twist angle is set to -45° here). Image changes with polarization state of incident light passing through films. The polarization states of the incident light are chosen to be left-handed circularly polarized (CP) light (left column), linearly polarized (middle column), and right-handed circularly polarized (CP) light (right column).

Supplementary Table 4. The comparison of the color gamut for the polarization dependent color switching.

structure	Color gamut	Light source	year, ref.
Al arcs	**14％-23％ NTSC**	circularly polarized light (CPL)	2019, 5
Porous nanocolumns	**~48％ NTSC**	linearly polarized (LP)	2020, 6
Cr-based plasma/LC	**50.4％ NTSC**	linearly polarized (LP)	2019, 7
PDA TSL films	**57％ NTSC**	circularly polarized light (CPL)	2021, 8
Heterostructure composite film	**184％ NTSC**	circularly polarized light (CPL)	This work

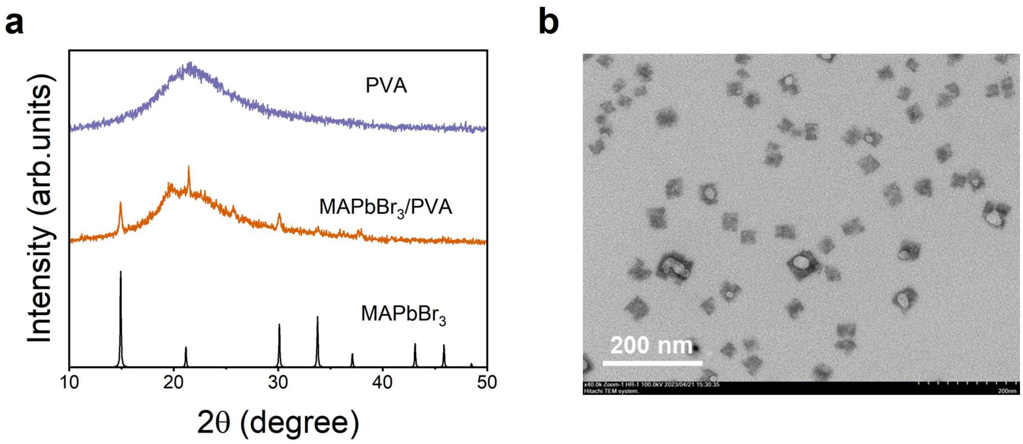

Supplementary Fig. 27 | Crystallographic result and the TEM of the $MAPbBr_3$ perovskite (**a**) XRD patterns of PVA and $MAPbBr_3$/PVA. (**b**) TEM images of $MAPbBr_3$/PVA.

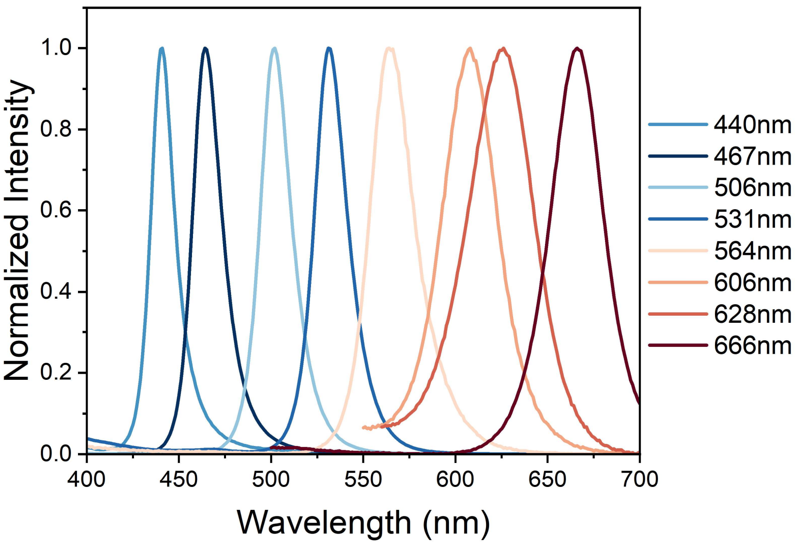

Supplementary Fig. 28 | Photoluminescence spectra characterization. The photoluminescence spectra of eight types of perovskite quantum dots (440 nm, 467 nm, 506 nm, 531 nm, 564 nm, 606 nm, 628 nm, 666 nm) were used in this study.

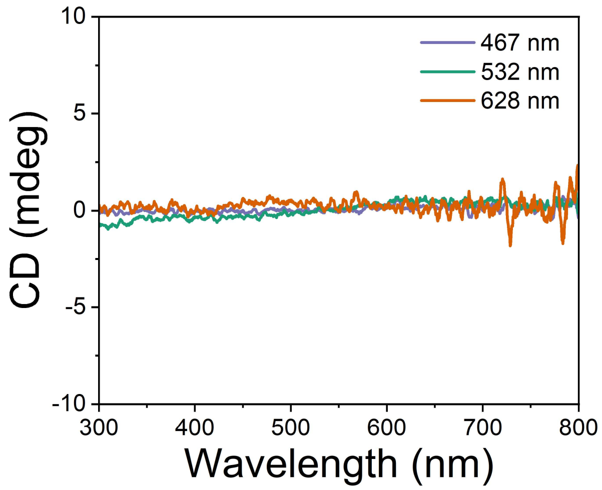

Supplementary Fig. 29 | CD spectra of the perovskite QDs. The CD spectra of blue (467 nm), green (532 nm) and red (628 nm) perovskite QDs.

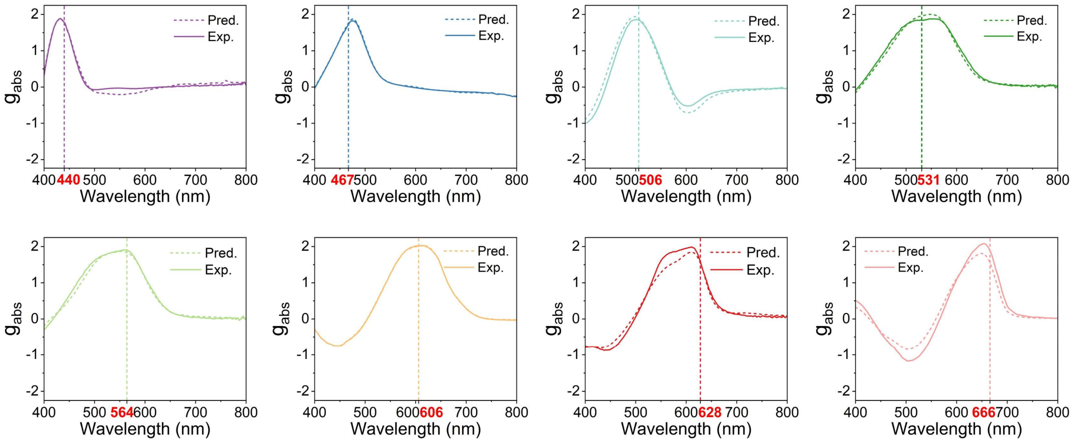

Supplementary Fig. 30 | Dissymmetry factor g*abs* spectra of eight chiroptical films used as filters to generate circularly polarized fluorescence. The dashed lines are the predicted curve, and the corresponding solid lines are the experimental ones.

Supplementary Table 5. The comparison of the luminescence dissymmetric factor g_{lum} for the perovskite based circularly polarized luminescence. (The absolute of the idea dissymmetry factor is 2).

structure	$\|g_{lum}\|$	method	year, ref.
$CsPbX_3$ NCs doped DGAm	**3×10^{-3}**	active	2018, 9
chiral molecules doped 2D perovskite	**0.176**	active	2019, 10
CLC/$CsPbX_3$/CLC	**1.6**	passive	2019, 11
Chiral perovskite-Liquid Crystals Bilayer	**1.9**	passive	2022, 12
Hetero-bilayer films/PQDs	**1.9**	passive	This work

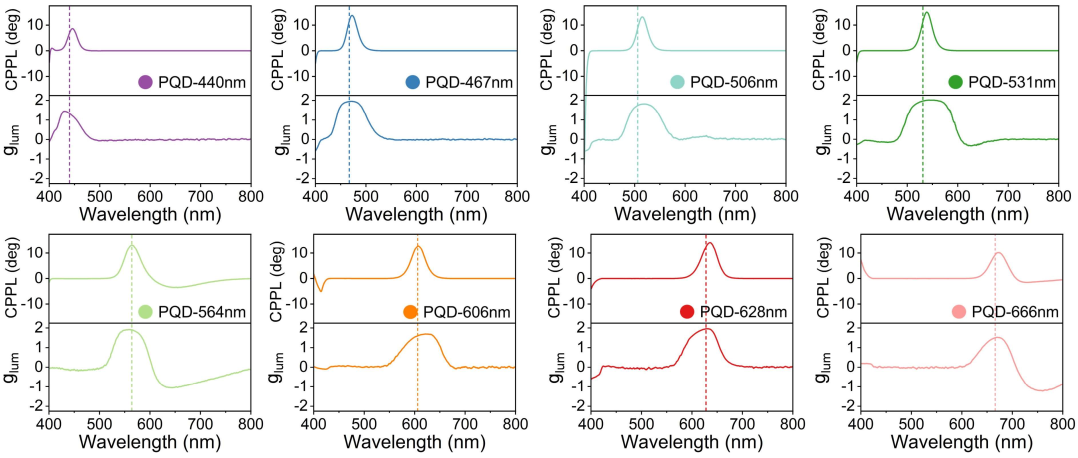

Supplementary Fig. 31 | The circularly polarized photoluminescence (CPPL) spectra of eight perovskite quantum dots. Experimental realization of inverse design of filters to maximize left-handed circularly polarized photoluminescence (LCPPL), instead of Right-handed circularly polarized photoluminescence (RCPPL) shown in the main text.

Supplementary Table 6. Quantum yield and figure of merit (FOM) of PQDs at 8 different wavelengths.

Wavelength	Φ	FOM
440 nm	0.023	0.03
467 nm	0.137	0.27
506 nm	0.128	0.25
531 nm	0.146	0.29
564 nm	0.115	0.22
606 nm	0.197	0.34
628 nm	0.211	0.41
666 nm	0.049	0.07

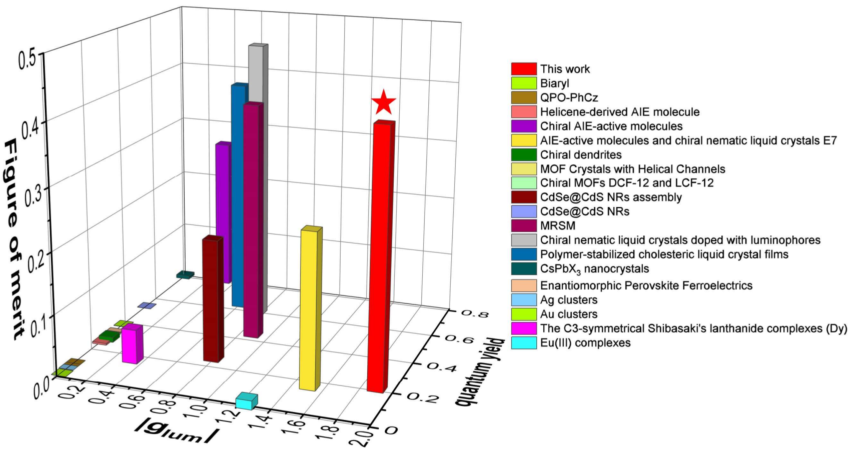

Supplementary Fig. 32 | Figure of merit (FOM) values for CPL materials. The comparison of FOM values between those in the literatures and those obtained in this work, and the data in this figure are taken from Supplementary Table 7.

Supplementary Table 7. Figure of merit (FOM) comparison of the CPL materials

Category	Materials	$\lvert g_{lum}\rvert$	Φ%	|FOM|	Ref.
Hetero-structured bilayer	Heterobilayer composite film/perovskite	1.9	21.1	0.41	This work
Small organic molecules	Biaryl	1×10^{-3}	39	3.9×10^{-4}	13
	QPO-PhCz	1.2×10^{-3}	10.6	1.272×10^{-4}	14
Molecular assemblies	Helicene-derived AIE molecule	0.011	25.6	2.8×10^{-3}	15
	Chiral AIE-active molecules	0.32	81.3	0.26	16
	Chiral AIE-active molecules and chiral nematic liquid crystals E7	1.51	16.56	0.25	17
	Chiral dendrites	0.03	28	8.4×10^{-3}	18
Metal organic frameworks (MOFs)	MOF Crystals with Helical Channels	1.15×10^{-2}	30	3.45×10^{-3}	19
	Chiral MOFs DCF-12 and LCF-12	2.5×10^{-3}	27.3	6.825×10^{-4}	20
Quantum dot with chiral ligands	CdSe@CdS NRs	0.8	25	0.2	21
	CdSe@CdS NRs	0.0005	54	2.5×10^{-4}	22
Chiral nematic liquid crystals as an optical filter	MRSM	0.89	44	0.39	23
	Chiral nematic liquid crystals doped with luminophores	0.77	60.4	0.465	24
	polymer-stabilized cholesteric liquid crystal films	0.61	64.2	0.392	25
Chiral Perovskite	$CsPbX_3$ nanocrystals	7.3×10^{-3}	80.7	5.6×10^{-3}	9
	Enantiomorphic Perovskite Ferroelectrics	6.1×10^{-3}	32.46	1.98×10^{-3}	26
Clusters	Ag clusters	1.2×10^{-3}	8	9.6×10^{-5}	27
	Au clusters	0.007	3.6	2.5×10^{-4}	28
lanthanide complexes	The C_3-symmetrical Shibasaki's lanthanide complexes (Dy)	0.33	17	0.0561	29
	Eu(III) complexes	1.25	1.16	1.45×10^{-2}	30

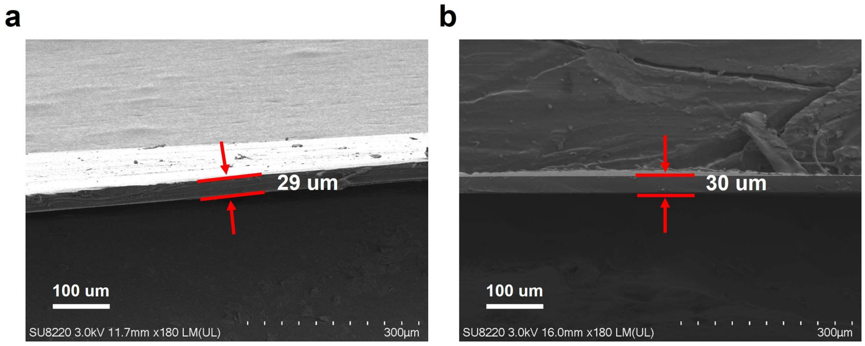

Supplementary Fig. 33 | SEM images of each layer in the stacked thin film. (**a**) Initial thickness of 48 μm and a stretching degree of 100% (transparent film). (**b**) Initial thickness of 80 μm dyed with dye7 and a stretching degree of 500% (dyed film).

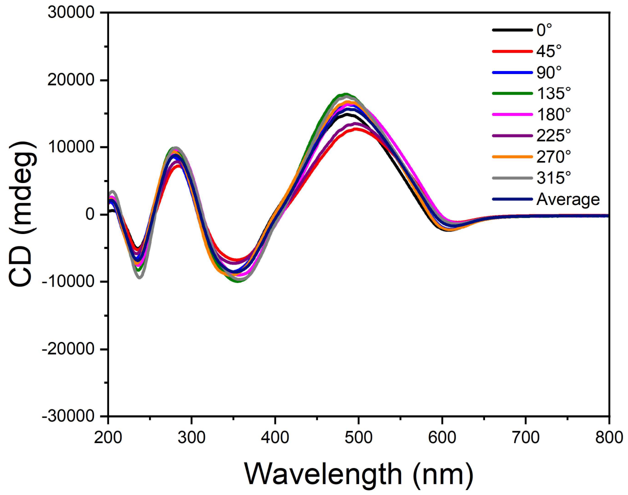

Supplementary Fig. 34 | Rotation stability of circular dichroism (CD) spectra of chiral films. The CD spectra of the chiral film rotated around the optical path at different angles and then averaged.

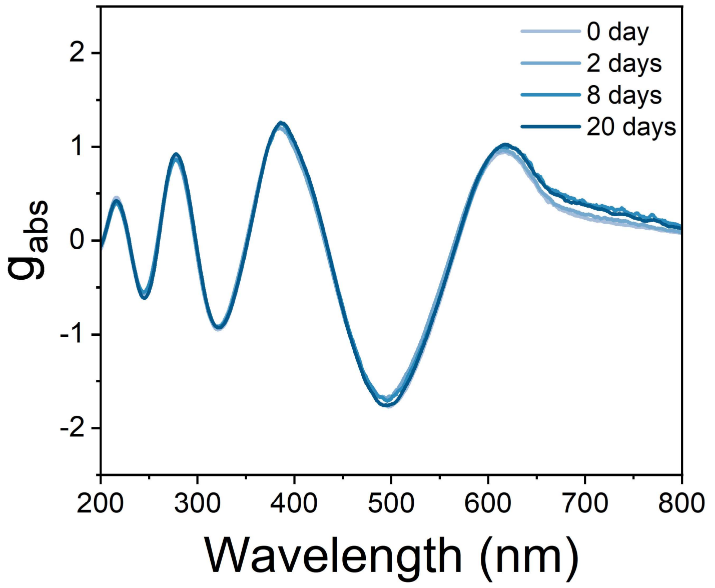

Supplementary Fig. 35 | The stability of the chiral film after treated with boric acid. The dissymmetry factor g_{abs} spectra of the chiral film before and after being exposed in air for 20 days.

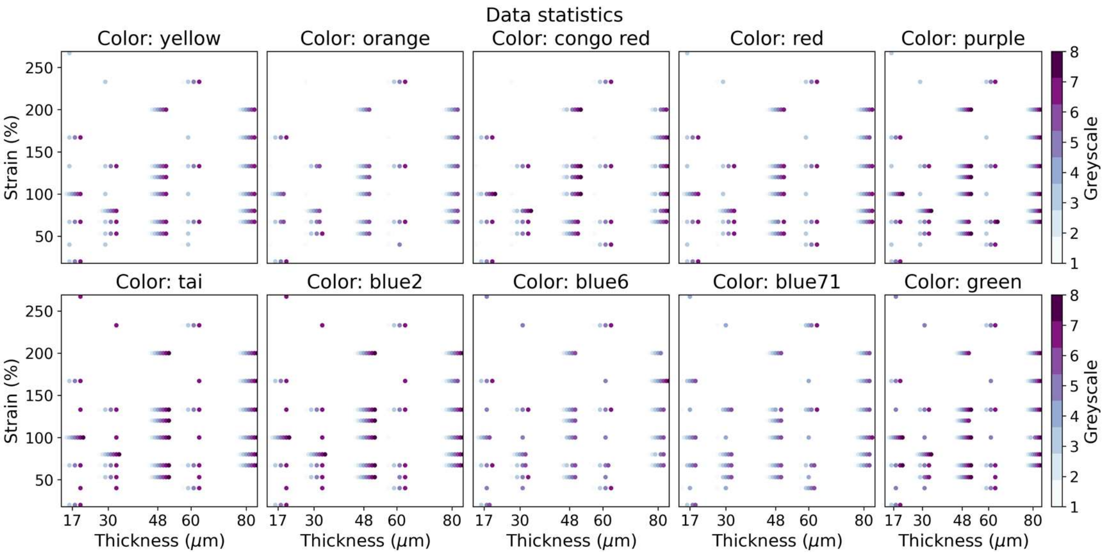

Supplementary Fig. 36 | The dataset of 1493 samples. The dataset of 1493 samples selected from the 10^5 possible combinations.

Supplementary References:

1 Wang, M. & Zhao, C. H. Chiral Triarylborane-Based Small Organic Molecules for Circularly Polarized Luminescence. *Chem. Rec.* **22**, e202100199 (2022).

2 Wang, Y. *et al.* Strong circularly polarized luminescence induced from chiral supramolecular assembly of helical nanorods. *Chem. Commun. (Camb)* **53**, 7505-7508 (2017).

3 Wan, L. *et al.* Inverting the Handedness of Circularly Polarized Luminescence from Light-Emitting Polymers Using Film Thickness. *ACS Nano* **13**, 8099-8105 (2019).

4 Lv, J. *et al.* Biomimetic Chiral Photonic Crystals. *Angew. Chem. Int. Ed. Engl.* **58**, 7783-7787 (2019).

5 Liu, H. L. *et al.* 3D chiral color prints for anti-counterfeiting. *Nanoscale* **11**, 5506-5511 (2019).

6 Ko, J. H., Yoo, Y. J., Kim, Y. J., Lee, S. S. & Song, Y. M. Flexible, Large-Area Covert Polarization Display Based on Ultrathin Lossy Nanocolumns on a Metal Film. *Adv. Funct. Mater.* **30**, 1908592 (2020).

7 Driencourt, L. *et al.* Electrically Tunable Multicolored Filter Using Birefringent Plasmonic Resonators and Liquid Crystals. *ACS Photon.* **7**, 444-453 (2019).

8 Xie, Y. *et al.* Flexible, Programmable, Chiroptical Polymer Films of Twisted Stacking Layers for Circular Polarization-Based Multiplex Color Display. *Adv. Opt. Mater.* **10**, 2102197 (2021).

9 Shi, Y., Duan, P., Huo, S., Li, Y. & Liu, M. Endowing Perovskite Nanocrystals with Circularly Polarized Luminescence. *Adv. Mater.* **30**, 1705011 (2018).

10 Ma, J. *et al.* Chiral 2D Perovskites with a High Degree of Circularly Polarized Photoluminescence. *ACS Nano* **13**, 3659-3665 (2019).

11 Wang, C. T. *et al.* Fully Chiral Light Emission from $CsPbX_3$ Perovskite Nanocrystals Enabled by Cholesteric Superstructure Stacks. *Adv. Funct. Mater.* **29**, 1903155 (2019).

12 Liu, S. *et al.* Circularly polarized perovskite luminescence with dissymmetry factor up to 1.9 by soft helix bilayer device. *Matter* **5**, 2319-2333 (2022).

13 Amako, T., Kimoto, T., Tajima, N., Fujiki, M. & Imai, Y. A comparison of circularly polarized luminescence (CPL) and circular dichroism (CD) characteristics of four axially chiral binaphthyl-2,2′-diyl hydrogen phosphate derivatives. *Tetrahedron* **69**, 2753-2757 (2013).

14 Yang, S.-Y. *et al.* Efficient circularly polarized thermally activated delayed fluorescence hetero-[4]helicene with carbonyl-/sulfone-bridged triarylamine structures. *J. Mater. Chem. C* **10**, 4393-4401 (2022).

15 Shen, C. *et al.* Helicene-derived aggregation-induced emission conjugates with highly tunable circularly polarized luminescence. *Mater. Chem. Front.* **4**, 837-844 (2020).

16 Liu, J. *et al.* What makes efficient circularly polarised luminescence in the condensed phase: aggregation-induced circular dichroism and light emission. *Chem. Sci.* **3**, 2737 (2012).

17 Yao, K. *et al.* Ultrastrong Red Circularly Polarized Luminescence Promoted from Chiral Transfer and Intermolecular Forster Resonance Energy Transfer in Ternary Chiral Emissive Nematic Liquid Crystals. *J. Phys. Chem. Lett.* **12**, 598-603 (2021).

18 Li, Q., Lu, X., Lv, Z., Zhu, B. & Lu, Q. Full-Color and Switchable Circularly Polarized Light from a Macroscopic Chiral Dendritic Film through a Solid-State Supramolecular Assembly. *ACS Nano* **16**, 18863-18872 (2022).

19 Zhang, C. *et al.* Enantiomeric MOF Crystals Using Helical Channels as Palettes with Bright White Circularly Polarized Luminescence. *Adv. Mater.* **32**, e2002914 (2020).

20 Gao, P. *et al.* Host-Guest Chemistry of Chiral MOFs for Multicolor Circularly Polarized

Luminescence Including Room Temperature Phosphorescence. *Adv. Funct. Mater.* 2300105 (2023).

21 Lv, J., Yang, X. & Tang, Z. Rational Design of All-Inorganic Assemblies with Bright Circularly Polarized Luminescence. *Adv. Mater.* **35**, e2209539 (2023).

22 Cheng, J. *et al.* Optically Active CdSe-Dot/CdS-Rod Nanocrystals with Induced Chirality and Circularly Polarized Luminescence. *ACS Nano* **12**, 5341-5350 (2018).

23 Guo, Q. *et al.* Multimodal-Responsive Circularly Polarized Luminescence Security Materials. *J. Am. Chem. Soc.* **145**, 4246-4253 (2023).

24 Zhang, W. *et al.* Circularly polarized luminescence from oriented polymer films doped with a tetraphenylethylene-based conjugated oligomer. *Mater. Chem. Front.* **5**, 5471-5477 (2021).

25 Yu, P. *et al.* Polymer-stabilized cholesteric liquid crystal films with broadband reflection formed by photomask polymerization. *Opt. Mater.* **136**, 113385 (2023).

26 Gao, J. X., Zhang, W. Y., Wu, Z. G., Zheng, Y. X. & Fu, D. W. Enantiomorphic Perovskite Ferroelectrics with Circularly Polarized Luminescence. *J. Am. Chem. Soc.* **142**, 4756-4761 (2020).

27 Zhang, M. M. *et al.* Alkynyl-Stabilized Superatomic Silver Clusters Showing Circularly Polarized Luminescence. *J. Am. Chem. Soc.* **143**, 6048-6053 (2021).

28 Shi, L. *et al.* Self-Assembly of Chiral Gold Clusters into Crystalline Nanocubes of Exceptional Optical Activity. *Angew. Chem. Int. Ed. Engl.* **56**, 15397-15401 (2017).

29 Deng, M., Schley, N. D. & Ung, G. High circularly polarized luminescence brightness from analogues of Shibasaki's lanthanide complexes. *Chem. Commun. (Camb)* **56**, 14813-14816 (2020).

30 Tan, Y. B. *et al.* Visible Circularly Polarized Luminescence of Octanuclear Circular Eu(III) Helicate. *J. Am. Chem. Soc.* **142**, 17653-17661 (2020).
