# Supplementary material for: Inverse design of chiral functional films by a robotic AI-guided system
Source: Nat Commun. 2023 Oct 4;14:6177. doi: 10.1038/s41467-023-41951-x (PMC10551020; doi:10.1038/s41467-023-41951-x)
Supplement: Supplementary file 3 — Description of Additional Supplementary Files [file 41467_2023_41951_MOESM3_ESM.pdf]

<u>Description of Additional Supplementary Files Document</u>

Supplementary Movie 1

Description: Overall workflow of AI-Chemist to construct films with optimal chiroptical performance.

Supplementary Movie 2

Description: Multiplex circular polarization-based laser display.
